# Revised Mimivirus major capsid protein sequence reveals intron-containing gene structure and extra domain

**DOI:** 10.1186/1471-2199-10-39

**Published:** 2009-05-11

**Authors:** Saïd Azza, Christian Cambillau, Didier Raoult, Marie Suzan-Monti

**Affiliations:** 1 Unité de Recherche sur les Maladies Infectieuses et Tropicales Emergentes (URMITE), CNRS-IRD UMR 6236, IFR 48, Faculté de Médecine, 27 Boulevard Jean Moulin, 13385 Marseille cedex 05, France; 2 Architecture et Fonction des Macromolécules Biologiques, UMR 6098, CNRS-Universités Aix-Marseille I et II, Campus de Luminy, Case 932, 163 Avenue de Luminy, 13288 Marseille cedex 09, France

## Abstract

**Background:**

*Acanthamoebae polyphaga Mimivirus *(APM) is the largest known dsDNA virus. The viral particle has a nearly icosahedral structure with an internal capsid shell surrounded with a dense layer of fibrils. A Capsid protein sequence, D13L, was deduced from the APM L425 coding gene and was shown to be the most abundant protein found within the viral particle. However this protein remained poorly characterised until now. A revised protein sequence deposited in a database suggested an additional N-terminal stretch of 142 amino acids missing from the original deduced sequence. This result led us to investigate the L425 gene structure and the biochemical properties of the complete APM major Capsid protein.

**Results:**

This study describes the full length 3430 bp Capsid coding gene and characterises the 593 amino acids long corresponding Capsid protein 1. The recombinant full length protein allowed the production of a specific monoclonal antibody able to detect the Capsid protein 1 within the viral particle. This protein appeared to be post-translationnally modified by glycosylation and phosphorylation. We proposed a secondary structure prediction of APM Capsid protein 1 compared to the Capsid protein structure of *Paramecium Bursaria Chlorella Virus *1, another member of the Nucleo-Cytoplasmic Large DNA virus family.

**Conclusion:**

The characterisation of the full length L425 Capsid coding gene of *Acanthamoebae polyphaga Mimivirus *provides new insights into the structure of the main Capsid protein. The production of a full length recombinant protein will be useful for further structural studies.

## Background

*Acanthamoebae polyphaga Mimivirus *was described for the first time in 2003 [1]. It was isolated from amoebae growing in water sample from a cooling tower during an outbreak of pneumonia in an English hospital. Compared to other members of the Nucleo-Cytoplasmic Large DNA Viruses (NCLDV) family [2], APM has very particular characteristics due to its size, structure, genome sequence [3], and replication cycle through a specific virus factory [4]. The virus particle was shown to be icosahedral, with a capsid shell diameter of 5000 Å covered by long fibers and appears to have at least two lipid membranes beneath its Capsid protein [5]. The APM 1.2-Mb genome encodes at least 911 proteins [Genbank: NC_006450] [6]. Proteomic analysis of proteins extracted from purified viral particles allowed the identification of 114 proteins including the Capsid protein D13L, coded by the L425 gene. The D13L Capsid protein was shown to be the most abundant and major glycoprotein of APM [7] and is thought to be the main component of the outermost protein shell layer. The protein sequence was deduced from the first available Methionine codon in the L425 open reading frame. Recently, based on mass spectrometry analysis of the Capsid protein D13L peptides, the original protein sequence was revised and completed with 142 supplementary N-terminal amino-acids (AA) [UniProtKB: Q5UQL7] [8] and thereafter named here Capsid protein 1. The aim of this study was to characterize the full length APM Capsid protein 1 coding gene and to provide new insights into the structure of the protein. Blast of the Capsid protein 1 coding sequence on the APM genome sequence revealed that the start codon might be located 2042 bp upstream from the start codon of the previously annotated L425 coding gene. We produced a recombinant full length Capsid protein 1 and specific monoclonal antibodies (mAb) that might be useful to develop further structural analysis or detection assays.

## Methods

### cDNA cloning and sequencing

Total RNA from uninfected or APM infected *A. polyphaga *was extracted using RNeasy extraction kit (Qiagen) as previously described [3]. The Capsid protein 1 cDNA was synthsesized from APM infected *A. polyphaga *RNA by using the Superscript One-Step RT-PCR with Platinum Taq kit (Invitrogen) with the following primers:

Q5UQL7NcoIF: 5'-GAAGGAGATATACCATGGCAGGTGGTTTACTCCAATTA-3' and Q5UQL7SmaIR: 5'-GATGAGAACCCCCCCCGGGATTACTGTACGCTAATCCG-3'. Underlined are the APM L425 gene specific sequences: nt 560 926 – 560 449 for the forward primer; nt 557 533 – 557 515 for the reverse primer. The resulting 1782 bp cDNA fragment was then cloned into the pIVEX 2.3 expression vector (Roche) at the NcoI and SmaI sites. Recombinant plasmids were selected, purified and then incorporated into the d-Rhodamine Terminator Cycle Sequencing Ready Reaction buffer kit with Amplitaq Polymerase FS (Applied Biosystems). Reaction products were resolved by using an ABI 3100 automated sequencer and sequence analysis was performed using the software package ABI Prism DNA Sequencing Analysis Software version 3.0 (Applied Biosystems). T7 promoter and T7 terminator primers were used as well as two primer pairs targeting internal regions of the cDNA: QUQL7-SF1: 5'-GCTGGCAGTAGTAATTCTGC-3'; QUQL7-SR1: 5'-GCAGAATTACTACTGCCAGC-3'; and QU5QL7-SF2: 5'-GAAGGTAATGATGGTAGAAG-3'; QU5QL7-SR2: 5'-CTTCTACCATCATTACCTTC-3'.

### RT-PCR analysis

RNA was extracted from uninfected or APM-infected amoebae at 0, 2, 4, 8 and 16 hours post infection. 100 ng of each RNA was submitted to RT-PCR amplification using the SuperScript One-Step RT-PCR kit (Invitrogen) with the Q5UQL7NcoIF/Q5UQL7SmaIR primer pair. cDNA synthesis was performed in one cycle of 30 minutes at 50°C, 3 minutes at 94°C and subsequent PCR reaction with 35 cycles of 30 seconds at 94°C, 30 seconds at 60°C, 30 seconds at 72°C, and one cycle of 10 minutes at 72°C. Amplified products were analysed by electrophoresis on 1% agarose gel. GeneRuler 1 kb DNA ladder (MBI-Fermentas) was used as a DNA size marker.

### Expression of the recombinant Capsid protein 1

Expression of the Capsid protein 1 was performed using a cell-free translation system [9], the High Yield RTS 500 *Escherichia coli *Circular Template kit (Rapid Translation System, Roche). Reactions were performed at 30°C for 24 h, with a stirrer speed of 120 rpm, with 15 μg of recombinant plasmid used as DNA template. The RTS sample was then centrifuged for 10 min at 10 000 rpm and the pellet was resuspended in Laemmli buffer, heated for 5 min at 95°C, resolved by 7.5% SDS-PAGE, followed by Coomassie blue staining or transfer onto nitrocellulose membrane for immunoblot analysis using a anti-Histidine monoclonal antibody. The recombinant Capsid protein 1 was extracted from polyacrylamide gels by the electroelution method using the ElutaTube™ Protein Extraction Kit (Fermentas Life Sciences) according to the manufacturer's protocol and used to immunise mice for the production of monoclonal antibodies.

### Monoclonal antibodies production

Three six week-old female BALB/c mice were inoculated three times intraperitoneally at 14-days interval with 2 μg electroeluted protein mixed with 400 μg aluminium hydroxide and 10 μg CpG as previously described [10]. Four days after the last immunisation, spleen cells fusion was performed with X63.Ag 8.653 myeloma cells (2:1) using 50% 1500 polyethylene glycol (Roche). Cells were grown in RPMI medium (Invitrogen) with 15% heat inactivated fetal calf serum (Invitrogen) and hypoxanthine-aminopterin-thymidine selective medium (Invitrogen) at 37°C with 5% CO2. Hybridoma supernatants were screened 10 days after by ELISA using plates coated overnight with 10 μg/ml of APM extract in sodium carbonate buffer 100 mM, pH 9.6. Positive hybridomas were subcloned by limiting dilution and submitted to isotyping using a mouse monoclonal isotyping kit (Sigma-Aldrich). Hybridomas producing the highest mAb titers were then subcloned, expanded, and tested by Western blot analysis using APM extract subjected to 2D gel electrophoresis and transferred onto nitrocellulose.

### 2D-gel electrophoresis

APM particles were purified through a 25% sucrose gradient and APM extract was prepared for 2-D gel electrophoresis as previously reported [7]. Immobiline™ DryStrips (7 cm, pH 3–10 GE Healthcare) were rehydrated overnight using 125 μl rehydration buffer [8 M urea, 2% (w/v) CHAPS, 60 mM DTT, 0,5% (v/v) IPG buffer (GE Healthcare)] containing 20 μg of solubilized APM proteins and IEF was carried out according to the manufacturer's protocol (IPGphor II, GE Healthcare). Before the second dimension electrophoresis was performed, strips were equilibrated twice in 5 ml equilibration buffer [30% (v/v) glycerol, 3% (w/v) SDS, 6 M urea, 50 mM Tris-HCl, bromophenol blue, pH 8.8] for 15 min. This buffer was supplemented with 65 mM DTT for the first equilibration and with 100 mM iodoacetamide for the second one. The strips were then embedded in 0.5% agarose and the proteins resolved by 10% SDS-PAGE (Mini-Protean III, Bio-Rad). Gels were stained either with silver or transferred onto nitrocellulose for Western blot analysis using Capsid protein 1 specific mAb; anti-Phosphothreonine, anti-Phosphotyrosine or anti-Phosphoserine (10^-4 ^dilution, Sigma) mAbs; or rabbit polyclonal antiserum anti-methylated Lysine (dilution 4.10^-2^, Biomol GmbH). The detection of glycosylated proteins in 2D gels was performed according to the Pro-Q Emeral 300 glycoprotein stain kit's procedure (Invitrogen) and visualized using a 300 nm UV illuminator.

## Results and discussion

**Figure 1:**
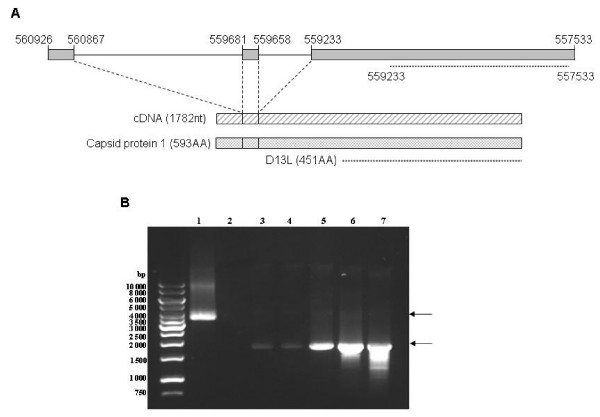
**Schematic organisation and expression of the full length L425 gene**. **(A) **L425 gene (nt 560926 – 557533 position on the APM genomic DNA) showed three coding sequences, boxed in grey, separated by two intron sequences. Organisation of the corresponding cDNA and 593AA full length protein are shown below. Locations of the former D13L capsid coding sequence and corresponding protein are indicated by dotted lines. (B) PCR on APM genomic DNA (lane 1) and RT-PCR analysis on RNA extracted from uninfected (lane 2) or APM-infected amoebae at 0, 2, 4, 8 and 16 h p.i. (lanes 3–7, respectively) using 5' Q5UQL7NcoIF and 3' Q5UQL7SmaIR primers. Left lane: DNA size marker.

The L425 gene (nt 557 530 – 558 885) was originally annotated as being the APM D13L Capsid protein coding sequence. Further mass spectrometry identification of D13L Capsid peptides allowed Suhre *et al*. to submit a protein sequence completed with 142 N-terminal AA [8]. Using TBLASTN [11] the coordinates of the corresponding nucleotide sequence on the APM genome appeared to be: nt 560 926 – 560 867 (coding AA 1–18); nt 559 681 – 559 658 (coding AA 19–27) and nt 559 233 – 557 533 (coding AA 27–593). This result suggested the existence of two untranslated regions: intron 1, 1188 bp long, (nt 560868 – 559 680) and intron 2, 427 bp long (nt 559 659 – 559 232) (Figure 1A).

Based on this sequence, outermost 5'- and 3'-terminal primers, Q5UQL7NcoIF and Q5UQL7SmaIR respectively, were designed to determine the Capsid protein 1 gene structure. RNA was extracted from APM-infected *A. polyphaga *and used to synthetise full length L425 cDNA. We then cloned the full-length cDNA into an expression vector and sequenced the target gene. Comparison of the cDNA sequence (1782 bp) with the APM genomic DNA sequence confirmed that the Capsid protein 1 gene is 3430 bp long and consists of three exons interrupted by two introns (Figure 1A). This result supposed that splicing events might occur during gene transcription and/or RNA maturation. To gain insight into this possibility, RT-PCR analyses were performed on RNA extracted at different time from APM-infected *A. polyphaga *using the Q5UQL7NcoIF/Q5UQL7SmaIR primer pair (Figure 1B). Agarose gel electrophoresis showed a fragment, about 4000 bp long, amplified from APM genomic DNA (lane 1), and a fragment, about 1800 bp long, from infected cell RNA (lanes 3–7), while no fragment was amplified from uninfected cell RNA (lane 2). Only fully spliced mRNA was detected from t0 to t16 p.i., with an increased signal. No precursor RNA could be identified. Detection of Capsid protein 1 mRNA as soon as t0 was not surprising since this RNA was shown to be packaged within the viral particle [3]. Additional RT-PCR experiments performed using primer pairs able to detect the potential different forms of spliced Capsid protein 1 RNA were unsuccessful to demonstrate alternative forms of spliced RNA whatever the time post-infection (data not shown).

**Figure 2:**
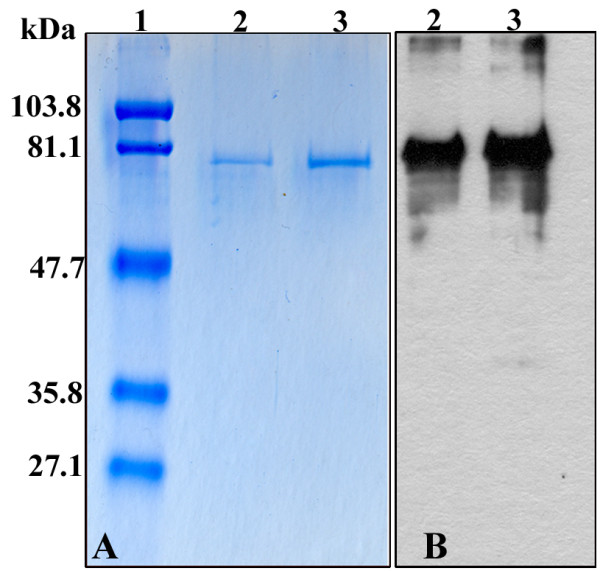
**Detection of the purified recombinant Capsid protein 1**. Either 0.5 or 1 μg of protein (lanes 2 and 3 respectively) was electrophoresed onto 10% SDS-PAGE and revealed by Coomassie blue staining (A) or Western-blot analysis using anti-Histidine mAb. Standard molecular weight markers (lane 1) are indicated on the left.

The full length 1782 bp Capsid protein 1 cDNA codes a 593 AA polypeptide with an estimated molecular mass of 67.27 kDa and theoretical isoelectric point of 5.27. The full length cDNA was cloned into the pIVEX 2.3 expression vector, producing a C-terminal His-tagged recombinant protein. Analysis of the purified recombinant protein revealed an apparent molecular weight of 69.61 kDa, in accordance with the calculated one (Figure 2). Expression of Capsid protein 1 was also successful in *E. coli *BL21 (DE3) pLysS. The protein was found in the unsoluble fraction and Western blot analysis using anti-His antibodies revealed the same pattern as with the in vitro translated protein (data not shown).

**Figure 3:**
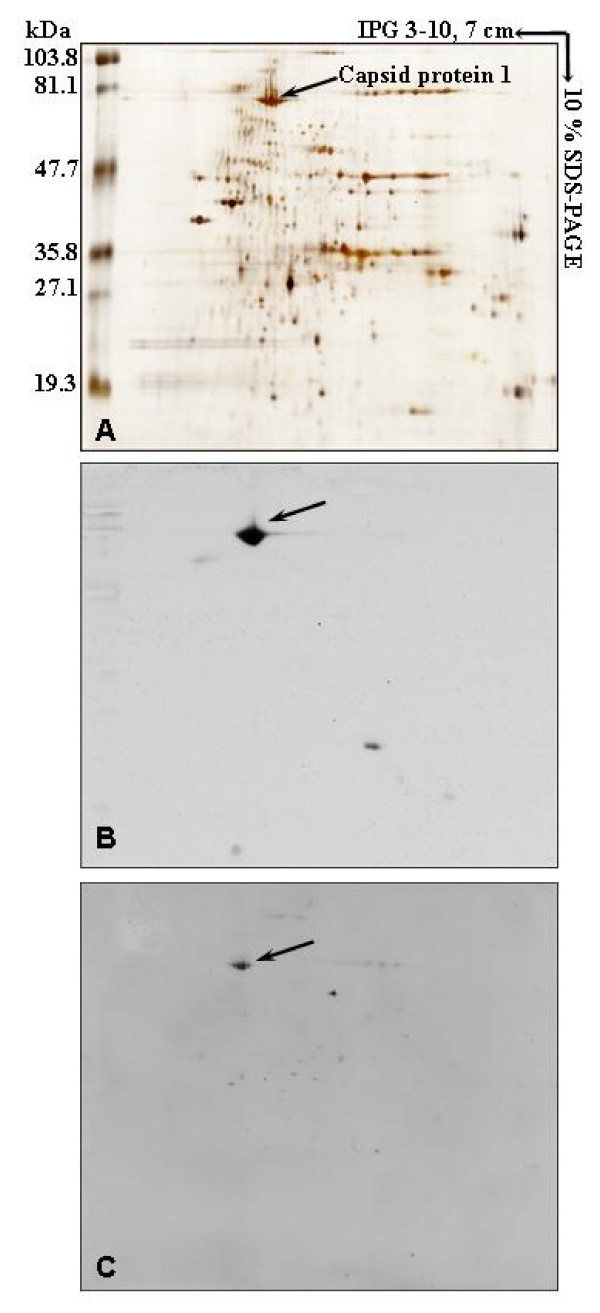
**Two-dimensional gel analysis of APM associated proteins**. Viral proteins were revealed by silver staining (A); by Western blot analysis using the Capsid protein 1 specific mAb P9A3 (B). Phosphorylated proteins were revealed with anti-Phosphoserine mAb (C). Standard molecular weight markers are indicated on the left.

Analysis of proteins extracted from purified APM particles, resolved by 2D gel electrophoresis, revealed after silver staining the typical electrophoresis pattern of APM proteins with the Capsid protein 1 appearing as four spots under silver staining with an apparent 68.95 kDa molecular weight (Figure 3A and [7]). Monoclonal antibodies (mAb) produced against the recombinant full length Capsid protein 1 were used for Western blot analysis of APM extract resolved by 2D gel analysis and transferred onto nitrocellulose membranes. The anti-Capsid protein 1 mAb P9A3 recognized the spots previously identified by mass spectrometry as the Capsid protein D13L (Figure 3B). It was previously shown that rabbit or mouse polyclonal antisera prepared against purified viral particles were unable to recognize any of these spots and that the D13L capsid protein was shown to be glycosylated [7]. APM Capsid protein 1 contains several Ser, Thr or Asn residues predicted to be N- or O-glycosylated, according to sequence analysis using the ExPASy proteomics server (data not shown) [12], but has no signal peptide. The absence of signal peptide suggests that APM Capsid protein 1 potential glycosylation sites are unlikely to be exposed to host cell glycosyltransferases. It has been shown in the PBCV1 Vp54 Capsid protein that N-glycosylation sites around Asn residues do not obey the consensus NX(T/S) motif, meaning that glycosylation may occur via the virus encoded glycosyltransferases rather by the host ones [13]. This may also be the case for APM Capsid protein 1. Other post-translational modifications were investigated such as AA phosphorylation or methylation. APM Capsid protein 1 appeared to be phosphorylated on Serine (Figure 3C), Threonine or Tyrosine residues but was not methylated (data not shown).

**Figure 4:**
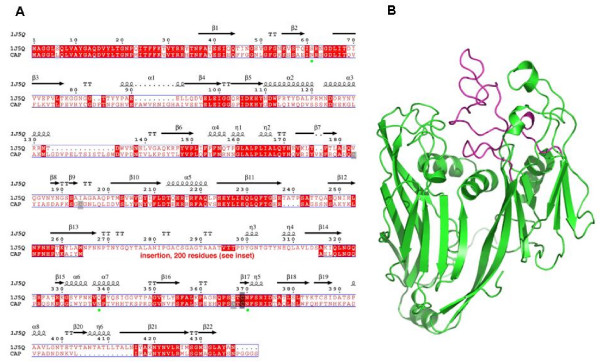
**Sequence alignment and 3D modelling of APM Capsid protein 1 with that of Chlorella virus PBCV1**. (A) Sequence numbering is from the Chlorella virus capsid [PDB:1J5Q]. The putative N-glycosylation sites in APM Capsid are identified by green circles. An insertion of 200 AA replaces a coil motif in 1J5Q (positions 265–315) not belonging to the «jelly-roll» motifs. (B) 3D structure representation of Chlorella virus PBCV1 Capsid core domain (green), and the coil domain (magenta) which is substituted in APM Capsid.

The former coding sequence assignment for the geneL425 was shown to be homologous to Vp54, the major Capsid proteins of *Paramecium Bursaria Chlorella Virus *1 (PBCV1) and other large DNA viruses [14]. The full length APM Capsid protein 1 expressed from the L425 gene exhibited even higher sequence identity with Vp54 with 46% identity in the N-terminal region (positions 1–287) and 40% in the C-terminal region (positions 483–593). The APM Capsid protein 1 is overall 45% identical to that of the Chlorella virus PBCV1 [PDB:1J5Q] [15] but 200 amino-acids are inserted at a position of a stretch of 50 residues in the Chlorella virus Capsid protein, between Trp264 and Ser315 (Figure 4A). The insertion has several distinct features: i) it is located at the external face of the APM Capsid protein 1; ii) it does not belong to the two «jelly roll» motifs of the core, as found in PBCV1 Capsid protein; iii) it replaces a group of 4 N-linked sugar, either because Asn residues are in the deletions/insertions sequence areas (3 cases) or are absent; and iv) it is predominantly structured as a motif of about 10 β-strands according to the PSIPRED protein structure prediction server [16-18]. Such a high β-strand contain is reminiscent of β-sandwich folds. The structure of PBCV1 Capsid is known [16] and is shown in Figure 4B. This structure is formed of two «jelly roll» motifs as found in many other viruses [14]. Compared to the PBCV1 Capsid structure, the APM CApsid protein 1 insertion might be very proeminent. It contains 4 Cysteine residues, on top of the 3 present in the core domain. It should be noticed that PBCV1 contains only 3 Cysteine residues, none of them involved in disulfide bridges. The presence of these conserved domains in the capsid proteins of large dsDNA viruses strengthens the hypothesis that the capsomer structure of APM should be similar to other large dsDNA icosahedral viruses where the capsid proteins assemble into a pseudo-hexameric capsomer organized into tri- and pentasymmetrons [19].

## Conclusion

Due to an erroneous gene model prediction, the coding sequence for L425 was wrongly assigned and was until now only partially described [3]. The full length coding gene appeared to be composed of three exons separated by two untranscribed introns. This leads to the synthesis of a full length 593AA protein translated from a spliced RNA. This feature seems to be unique to APM since the PBCV1 A383R or the ASFV B646L capsid coding genes do not contain intron sequence [20,21]. Tentative bioinformatics analyses of the APM L425 gene introns provided poor informations about their sequences. Intron 1 (nt 560868 – 559 680) matched with an evalue of 1e-4 to group I introns while being not related to a specific subgroup [22,23]. Intron 2 (nt 559 659 – 559 232) did not show significant match with either group I or group II introns [22]. Until now, the only other described introns in APM genome are self-splicing introns present in the coding sequences of the two largest RNA polymerase subunits coding genes L244 and R501 [3].

Rabbit or mouse immune sera produced against purified APM particles were unable to recognize the Capsid protein as an antigen in intact viruses, most probably due to the presence of a dense layer of fibrils surrounding the capsid shell. The availability of a monoclonal antibody against Capsid protein 1 might be useful for the development of detection assays in clinical samples since APM might represent a novel human pathogen [24-26].

APM Capsid protein 1 is a glycosylated protein. However its sequence contained no signal peptide, which makes the potential glycosylation sites unlikely to be exposed to the cellular glycosylation machinery. Post-translation modifications might occur in the virus factory since viral proteins appeared to be synthesised herein [3,4]. It might be thought that APM possibly use alternative pathways for translation and post-translational modifications. Metabolic studies on APM infected amoebae will contribute to understanding the complex interactions between host and pathogen. The recombinant full length Capsid protein might also represent a helpful tool to determine the structural organisation of APM viral capsid.

Annotation of APM genome revealed 1262 putative ORFs of length = 100 amino acid residues. 911ORFs were predicted to be protein coding genes and 298 of them had functional attributes [3]. Description of the full length Capsid coding gene and characterisation of the corresponding protein demonstrated that structural and functional studies will contribute to improve our knowledge of gene composition and expression of such a complex genome.

## Authors' contributions

SA performed the experimental work; CC contributed structure prediction and analysis; MSM and DR conceived the study and wrote the paper. All authors read and approved the final manuscript.
